# Time-specific lipid and gene expression responses to chilling stress in panicoid grass

**DOI:** 10.1093/jxb/erae336

**Published:** 2024-09-26

**Authors:** Artik Elisa Angkawijaya

**Affiliations:** RIKEN Center for Sustainable Resource Science, Yokohama, Japan

**Keywords:** Chilling stress, daily rhythmicity, lipid metabolism, panicoid grasses

## Abstract

This article comments on:

**Kenchanmane Raju SK, Zhang Y, Mahboub S, Ngu DW, Qiu Y, Harmon FG, Schnable JC, Roston RL.** 2024. Rhythmic lipid and gene expression responses to chilling in panicoid grasses. Journal of Experimental Botany **75**, https://doi.org/10.1093/jxb/erae247

This article comments on:

**Kenchanmane Raju SK, Zhang Y, Mahboub S, Ngu DW, Qiu Y, Harmon FG, Schnable JC, Roston RL.** 2024. Rhythmic lipid and gene expression responses to chilling in panicoid grasses. Journal of Experimental Botany **75**, https://doi.org/10.1093/jxb/erae247

**Cellular membranes are dynamic structures that enable plants to adapt to environmental changes, including extreme temperatures, by altering their membrane lipid composition and the saturation level of acyl groups. Plants modify their membrane fluidity and cellular integrity in response to chilling stress. While tropical-origin plants are prone to chilling stress, others within the same family exhibit better tolerance. Yet, no consistent changes of lipid abundance have been reported across different species. Kenchanmane Raju *et al*. (2024) studied panicoid grasses with varying levels of chilling tolerance, identifying conserved lipid changes and distinct time-specific responses in chilling-tolerant foxtail millet, similar to those observed in *Arabidopsis thaliana*. By correlating lipid and gene expression data, they identified multiple genes likely to be responsible for changing lipid responses at specific times of day during chilling in the tolerant foxtail millet with respect to the chilling-susceptible sorghum.**

## Impact of chilling stress on plant growth and lipid adaptations

Chilling stress, which occurs at low, non-freezing temperatures, is a major challenge for plant growth and development, ultimately threatening crop yield and food security. Tropical-origin crops such as maize (*Zea mays*), sorghum (*Sorghum bicolor*), and rice (*Oryza sativa*) are susceptible to chilling stress. They exhibit various chilling injury symptoms including delayed germination, reduced photosynthetic carbon assimilation, and adverse effect on plant reproduction (Allen and Ort, 2001; Zinn *et al.*, 2010; Zhang *et al.*, 2017). Just like in other organisms, membranes of plants encounter rigidification with decreasing temperatures, which increases their susceptibility to damage (Los and Murata, 2004; Zoldan *et al.*, 2012). Interestingly, the impact of chilling temperatures varies between tropical and temperate plants. Plants from temperate regions such as the winter biotype of Camelina (Joelle), wheat (*Triticum aestivum*), and Arabidopsis (*Arabidopsis thaliana*) show better tolerance to chilling temperatures (Cook *et al.*, 2004; Anderson *et al.*, 2022; Zou *et al.*, 2023).

Extensive studies on lipid metabolism of cold-acclimated *A. thaliana* have demonstrated that alteration in membrane lipids (including abundance, composition, and saturation status) is one of the adaptive responses to cope with chilling stress (Routaboul *et al.*, 2000; Welti *et al.*, 2002; Vaultier *et al.*, 2006). However, studies on these lipid changes have been conducted at varying periods and temperatures. Some studies focused on reporting the change of lipids during freezing temperatures (below 0 °C), while others included both cold acclimation and freezing stress. Given the nature of chilling stress, data reported on cold acclimation are more suitable for describing lipid alteration during chilling stress, as plants face prolonged low, non-freezing temperatures during this condition. Welti *et al*. (2002) reported the membrane lipid profile in 35-day-old Arabidopsis plants with cold exposure to 4 °C for 3 d, noting a significant increase in major glycerolipids monogalactosyldiacylglycerol (MGDG) and digalactosyldiacylglycerol (DGDG), and an average increase in phosphatidylethanolamine (PE) and phosphatidylcholine (PC), although the latter was not statistically significant. These findings were consistent with earlier reports by Uemura *et al*. (1995). Additionally, increases were observed in polyunsaturated glycerolipids, including 34:6-MGDG, 36:6-DGDG, 36:5-PC, 36:6-PC, 36:5-PE, and 36:6-PE, along with the significant decrease in saturated glycerolipid species (Uemura *et al.*, 1995; Welti *et al.*, 2002). These changes in lipid composition are crucial for chloroplast biogenesis and maintenance of photosynthetic activity during growth at low temperatures (Routaboul *et al.*, 2000), and to increase the freezing tolerance by reducing or eliminating the tendency of cellular membranes to form rigid non-bilayer structures when exposed to freezing temperature (Uemura *et al.*, 1995).

Advancing from lipid metabolic changes, more recent studies have identified possible conserved responses in different plant species when exposed to low-temperature stress, with consistent up-regulation of C-repeat binding factors (CBFs) and down-regulation of photosynthesis- and chloroplast-related genes (Kenchanmane Raju *et al.*, 2018; Lee *et al.*, 2023). Yet, reports on changes in lipid content and composition in response to cold stress across land plants have been inconsistent, probably due to genetic and physiological differences, as well as variations in experimental designs and sampling times (Kenchanmane Raju *et al.*, 2018). Furthermore, the lack of conservation in chilling adaptation among subfamilies of *Pooideae* grass suggests that different plant lineages have adapted to different temperate environments using distinct genetic and physiological mechanisms (Zhang *et al.*, 2017).

## Comparative study on chilling tolerance in panicoid grasses

To elucidate the genetic and lipid metabolic responses that contribute to chilling tolerance, Kenchanmane Raju *et al.* (2024) conducted a comprehensive study on panicoid grasses with varying levels of tolerance to chilling stress, focusing on foxtail millet (*Setaria italica*) and chilling-susceptible sorghum and Urochloa (*Urochloa fusca*). The 24 h dataset on lipid changes during the stress treatment revealed species-specific lipid abundance and unsaturation.

**Fig. 1.**
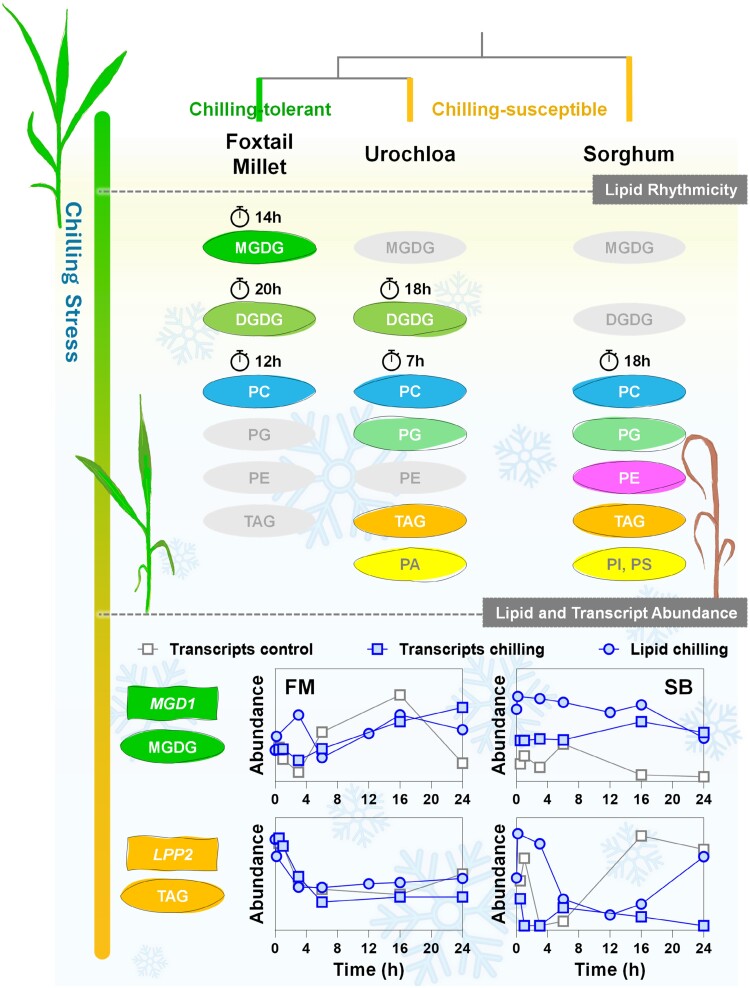
Global lipid oscillatory profile in chilling-induced panicoid grasses and representative transcript expression and lipid abundance correlation responsible for chilling tolerance in foxtail millet. Data obtained from Kenchanmane Raju *et al.* (2024) and visualized using GraphPad Prism 10.0. Metabolites and genes in colour are rhythmic, while those in pale grey are non-rhythmic.

Foxtail millet, known for its chilling tolerance, exhibited distinct rhythmicity in its major glycerolipids (MGDG, DGDG, and PC), with significant MGDG accumulation at the end of 24 h of chilling stress (Fig. 1). This MGDG rhythmicity and accumulation were absent in the other studied panicoid grasses. Urochloa, genetically close to foxtail millet, displayed similar lipid rhythmicity (DGDG and PC). Both species demonstrated increased abundance of DGDG between 18 h and 20 h upon chilling stress, suggesting a genetic influence on lipid response to chilling stress. This DGDG rhythmicity was not observed in sorghum. Notably, the rhythmicity of PC was conserved across all grasses studied, including sorghum, underscoring a common adaptive trait among these species. In addition to changes in glycerolipid abundance, foxtail millet displayed a significant early increase in the unsaturation of DGDG, phosphatidylglycerol (PG), phosphatidylserine (PS), and sulfoquinovosyldiacylglycerol (SQDG) compared with Urochloa and sorghum, indicating an early response to chilling stress. However, the unsaturation index of total lipid in all grasses did not show any significant differences over the 24 h chilling treatment. The rhythmicity of lipid changes during chilling stress in foxtail millet was similar to that observed in Arabidopsis, particularly in terms of MGDG abundance upon chilling or cold acclimation (Welti *et al.*, 2002; Kenchanmane Raju *et al.*, 2024), although the amplitude of diel variations in lipidome composition was less pronounced compared with grasses (Kenchanmane Raju *et al.*, 2024).

The study also explored the species-specific differences in chilling adaptation by examining the relationship between lipid-related gene expression and lipid abundance profiles. In foxtail millet, lipid phosphate phosphatase 2 (*LPP2*), possibly encoding a phosphatidic acid phosphatase, showed a significant correlation with the accumulation of triacylglycerol (TAG) during chilling stress, indicating that phospholipids serve as the primary source of TAG in foxtail millet when responding to chilling conditions. This correlation was not found in the sorghum orthologue, *SbLPP2*, highlighting a distinct difference in how these two species manage membrane lipid conversion to TAG under stress. Moreover, the authors proposed an important role for non-specific phospholipase C1 (*NPC1*) in the accumulation of PE and overall chilling tolerance in panicoid grasses. The 24 h data series clarifies why previous studies struggled with finding consistent lipid patterns among species and emphasizes the importance of considering time-specific changes to elucidate lipid metabolism under chilling stress (Kenchanmane Raju *et al.*, 2024).

## Circadian clock gating in response to chilling stress

In Arabidopsis and other seed plants, rhythmic changes in glycerolipid composition and their saturation status have been well documented (Browse *et al.*, 1981; Ekman *et al.*, 2007; Maatta *et al.*, 2012; Nakamura *et al.*, 2014). Extending this knowledge, a time-course data analysis in panicoid grasses confirmed the occurrence of oscillatory profiles in their lipid data, again highlighting the importance of time-specific data in evaluating the contribution of the circadian clock in regulating stress responses (Kenchanmane Raju *et al.*, 2024). Several review articles have compiled ample evidence of correlation between the circadian clock and various stresses, including cold/chilling stress (Dong *et al.*, 2011). The circadian clock enables plants to anticipate and prepare for environmental stresses that exhibit diurnal or seasonal oscillations, such as temperature fluctuations between day and night, as well as prolonged stress conditions (Lee and Thomashow, 2012; Verslues *et al.*, 2022). While it has been suggested that clock genes regulate the expression of lipid-related genes and CBF-encoding genes, future investigations should explore the potential feedback regulation. Specifically, research should focus on how disruption of the CBF pathway and altered lipid compositions affect the circadian clock. Additionally, although *CBF* genes have been identified in both Arabidopsis and sorghum, little is known about the lipid profiles in plants with loss of CBF function under normal or chilling-stressed conditions.
